# Surgical resection versus biopsy in the treatment of primary central nervous system lymphoma: a systematic review and meta-analysis

**DOI:** 10.1007/s11060-022-04200-7

**Published:** 2022-11-30

**Authors:** Rafał Chojak, Marta Koźba-Gosztyła, Karolina Polańska, Marta Rojek, Aleksandra Chojko, Rafał Bogacz, Natalia Skorupa, Jakub Więcław, Bogdan Czapiga

**Affiliations:** 1 Faculty of Medicine, Wroclaw Medical University, Ludwika Pasteura 1, 50-367 Wrocław, Poland; 2 Department of Neurosurgery, 4th Military Hospital in Wroclaw, Wrocław, Poland; 3 Department of Nervous System Diseases, Faculty of Health Sciences, Wroclaw Medical University, Wrocław, Poland

**Keywords:** PCNSL, Surgery, Biopsy, Hazard ratio, Overall survival, Progression-free survival

## Abstract

**Purpose:**

Despite the improvement in treatment and prognosis of primary central nervous system lymphoma (PCNSL) over the last decades, the 5-year survival rate is approximately 30%; thus, new therapeutic approaches are needed to improve patient survival. The study’s aim was to evaluate the role of surgical resection of PCNSL.

**Methods:**

Primary outcomes were the overall survival (OS) and progression-free survival (PFS) of patients with PCNSL who underwent surgical resection versus biopsy alone. The meta-analysis was conducted to calculate pooled hazard ratios (HRs) under a random-effects model for the time-to-event variables. The odds ratios (ORs) were calculated for binary, secondary outcome parameters.

**Results:**

Seven studies (n = 1046) were included. We found that surgical resection was associated with significantly better OS (HR 0.63 [95% CI 0.51–0.77]) when compared with biopsy. PFS was also significantly improved (HR 0.64 [95% CI 0.49–0.85]) in patients who underwent resection compared with those who underwent biopsy. The heterogeneity for OS and PFS was low (I2 = 7% and 24%, respectively). We also found that patients who underwent biopsy more often had multiple (OR 0.38 [95% CI 0.19–0.79]) or deep-seated (OR 0.20 [95% CI 0.12–0.34]) lesions compared with those who underwent surgical resection. There were no significant differences in chemotherapy or radiotherapy use or the occurrence of postoperative complications between the two groups.

**Conclusion:**

In selected patients, surgical resection of PCNSL is associated with significantly better overall survival and progression-free survival compared with biopsy alone.

**Supplementary Information:**

The online version contains supplementary material available at 10.1007/s11060-022-04200-7.

## Introduction

Primary central nervous system lymphoma (PCNSL) is an aggressive entity that accounts for approximately 6.7% of all CNS tumors and has an incidence of 0.45 per 100,000 person-years [1]. The majority of PCNSLs are diffuse large B-cell lymphomas [2–6]. The disease primarily affects the elderly [1, 7–9], and those with compromised immune systems [7, 8]. The most common symptoms that develop over weeks are focal neurologic deficits, followed by mental status and behavioral changes, increased intracranial pressure, and seizures [10]. Rarely do patients exhibit so-called B symptoms (frequently present in other lymphomas) such as fever, weight loss, or night sweats [11]. PCNSL is most commonly a single, supratentorial brain lesion [10]. The most typical locations are the frontoparietal lobe, followed by the temporal lobe, basal ganglia, and corpus callosum [10, 12, 13]. Less frequent locations include the cerebellum and brainstem; approximately 1% of patients have spinal cord involvement [12, 13].

PCNSL is diagnosed based on MRI, followed by a subsequent biopsy to establish histopathological confirmation before treatment [14–16]. Contrary to other brain tumors, PCNSL often responds well to chemotherapy. However, the disease is fatal if untreated [17]. Therefore, once the diagnosis is achieved, high-dose methotrexate-based (HD-MTX) chemotherapy is recommended as a first-line treatment for PCNSL [15, 18]. Almost a third of patients with PCNSL are primary refractory to first-line treatment, and the disease often relapses in treatment responders [19, 20]. Overall, the prognosis for patients with PCNSL is poor, with a 5-year survival rate of approximately 28% [7, 21, 22].

In the past, surgery for PCNSL was usually contraindicated [23] (some exceptions were large lesions that caused increased intracranial pressure and immediate symptoms of brain herniation [15]). In 2012, Weller et al. [24] challenged the traditional view that the extent of resection has no prognostic impact on patients with PCNSL and proposed reconsideration of the dogma that resection for PCNSL should be discouraged in every case. Since the study by Weller et al., several studies have reported that surgical resection might be beneficial for some patients [3, 25, 26]. However, studies reporting that resection has no benefit in terms of OS or PFS have also been published [27].

Overall, the role of surgical resection in the treatment of PCNSL is unclear, and as of today, there is no clear consensus about whether to recommend resection or biopsy for PCNSL patients [3, 18].

The main aim of this meta-analysis was to compare the overall survival and progression-free survival of patients with PCNSL who underwent surgical resection versus those who underwent biopsy alone.

## Methods

### Overview

The meta-analysis was performed in accordance with the preferred reporting items for systematic reviews and meta-analyses (PRISMA) guidelines and recommendations [28].

### Search strategy

We performed an electronic search of articles reporting data on surgical resection of PCSNL. We searched four databases (PubMed, Embase, Web of Science, and Scopus) from 2001 to October 1, 2022. The search syntax is presented in Online Appendix 1.

All titles and abstracts were independently reviewed for suitability by four researchers (MR, AC, RB, and JW). The full texts of potentially relevant articles were retrieved in order to perform a thorough eligibility analysis based on the selection criteria. Any discrepancies during the selection and extraction processes were resolved by discussion and consensus.

### Selection criteria

We included all English-language articles that compared resection with biopsy for patients with PCNSL and provided hazard ratios (HRs) for the primary outcomes or sufficient data to calculate them. The criteria for excluding studies were: (1) a sample size of less than 10 patients per study arm; (2) studies that used data from national databases; (3) reviews, case studies, conference abstracts, and letters to the editor; and (4) studies with irrelevant data. All studies that missed key data regarding survival for patients who underwent resection or biopsy, contained non-extractable data, or had data that might have potentially overlapped have also been excluded.

### Outcomes

The primary time-to-event outcomes of interest were overall survival (OS) and progression-free survival (PFS). Secondary outcomes included deep location of the lesion, multiple lesions, chemotherapy and radiotherapy treatment, and postoperative complications.

### Data extraction

Four researchers (MR, AC, RB, and JW) independently extracted data from included articles into a spreadsheet using Microsoft Excel (2010; Microsoft Corporation, Redmond, WA, USA). We recorded: the first author’s last name; year of publication; enrollment dates; country of a study performed; numbers of patients with PCNSL; sex; age; hazard ratios (HRs) and 95% CIs of death and progression among patients undergoing resection compared with biopsy; numbers and location of lesions; treatment (chemotherapy, radiotherapy); and complications (as defined in each study).

### Quality assessment

We used the Newcastle–Ottawa Scale [29] to assess the quality of the included studies. Two reviewers (KP and RC) carried out quality assessments individually. Any discrepancies were discussed by both authors. The NOS ranges from zero (highest risk of bias) up to nine points (lowest risk of bias). Studies with scores of ≥ 6 were considered high-quality.

### Statistical analysis

The meta-analysis was conducted to calculate pooled hazard ratios (HRs) and the corresponding 95% confidence intervals under a random-effects model for the time-to-event variable. Whenever possible, we obtained the HR and its 95% confidence interval (CI) from included studies. Otherwise, Kaplan–Meier (KM) plots were used for the indirect estimation of HR and its variance. KM plots were digitized to obtain survival probabilities and follow-up times using an Engauge digitizer (Free Software Foundation, Inc., Boston, USA). The extracted data were entered into the spreadsheet developed by Sydes and Tierney [30], which was then used to reconstruct the HR, ln[HR], and its corresponding se(ln[HR]). The odds ratios (ORs) were calculated for binary, secondary outcome parameters. The difference in age between patients who had resection versus biopsy was calculated based on available data according to the quantile estimation method [31]. We employed the Cochran’s Q test and the I2 statistic, which measures the proportion of overall variation among studies, to assess heterogeneity. Considerable heterogeneity is indicated by a Cochran’s Q P value of < 0.10. A significant amount of heterogeneity is indicated by an I2 statistic value greater than 50% [32]. We performed a leave-one-out analysis to assess the robustness of the main estimates and, in case of significant heterogeneity, investigate which study contributed to the heterogeneity the most. A value of P less than 0.05 was considered significant. Results for primary outcomes are presented in a forest plot with a 95% CI. All analyses were done in RStudio (version 1.3.1093).

## Results

### Search results

**Fig. 1:**
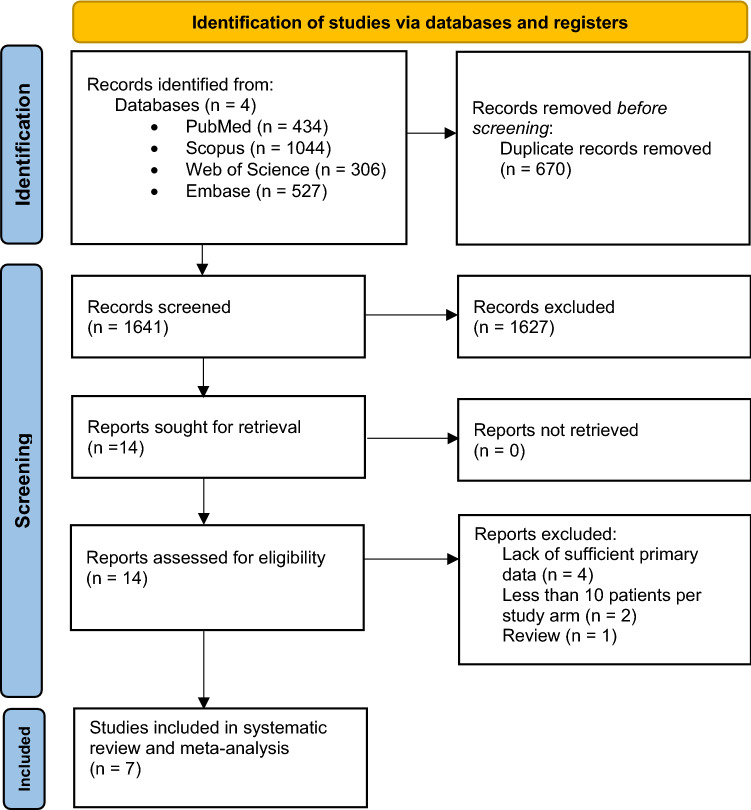
Flow chart showing search strategy. *From* Page MJ, McKenzie JE, Bossuyt PM, Boutron I, Hoffmann TC, Mulrow CD, et al. The PRISMA 2020 statement: an updated guideline for reporting systematic reviews. BMJ 2021;372:n71. 10.1136/bmj.n71. For more information, visit: http://www.prisma-statement.org/

By searching the database, we found 2311 records; 670 duplicates were removed. Of these, the suitability of 14 full-text articles was evaluated. Finally, seven studies [3, 4, 24, 25, 27, 33, 34] were included. Figure 1 shows the study identification process.

Two studies were from Europe, two were from Asia, one was from North America, and one was from South America. One study included patients from both Europe (Italy) and Asia (Israel). Five studies were retrospective case series (four single-center, one double-center). One study was a single-center prospective case series, and the other was a post hoc analysis of prospectively collected data from 75 German centers. The quality of the included studies ranged from 5 to 7, with a mean score of 6.4 (SD: 0.9). Table 1 summarizes the included studies, population characteristics, and quality assessment.

**Table 1:** Summary of included studies

Study	Country	Study design	NOS	Study period	Biopsy			Resection		
					N patients (%)	Age*	Male, N (%)	N patients (%)	Age*	Male, N (%)
Villalonga et al. [33]	Argentina	R	7	1994–2015	29 (62)	61	16 (55)	18 (38)	59	14 (78)
Schellekes et al. [3]	Israel, Italy	R	7	2005–2019	77 (68)	65	40 (52)	36 (32)	61	14 (39)
Wu et al. [25]	China	R	5	2013–2019	42 (60)	57	22 (52)	28 (40)	48	18 (64)
Jahr et al. [27, 36]	Norway	P	7	2003–2014	47 (59)	NR	NR	32 (41)	NR	NR
Ouyang et al. [4]	China	R	5	2009–2018	18 (20)	NR	NR	71 (80)	NR	NR
Weller et al. [24]	Germany	PH	7	2000–2009	379 (73)	63	NR	137 (27)	62/63	NR
Rae et al. [34]	USA	R	7	2000–2017	72 (54.5)	67	35 (49)	60 (45.5)	63	27 (45)

*R* retrospective, *P* prospective, *PH* post-hoc analysis of prospectively collected data, *NOS* Newcastle–Ottawa Scale, *NR* not reported

*Median or mean (as reported in study)

### Clinical and tumor features

Resection of the tumor was performed in 382 patients; 664 patients had a biopsy alone. Patients who underwent biopsy were older and more often had multiple, deep-seated lesions compared with patients who underwent resection. We found no significant differences in the use of chemotherapy, radiotherapy, or the occurrence of postoperative complications between the resection group and the biopsy group. These results are presented in Table 2.

**Table 2:** Meta-analysis of clinical and tumor features data of resection versus biopsy in patients with PCNSL

	No. of studies (No. of total patients)	No. of analyzed patients (resection/biopsy)	No. of events (resection/biopsy)	OR (95% CI)	I2 (%) (P value)
Deep location	4 (362)	142/220	35/127	0.20 (0.12–0.34)	0% (0.51)
Multiple lesion	4 (673)	217/456	58/209	0.38 (0.19–0.79)	51% (0.10)
B-cell lymphoma	3 (292)	114/178	106/164	1.32 (0.53–3.29)	0% (0.80)
Radiotherapy	3 (315)	66/118	29/47*	0.88 (0.45–1.71)	0% (0.65)
Chemotherapy	4 (831)	216/515	206/499**	0.90 (0.38–2.13)	0% (0.76)
Complications	2 (183)	64/119	9/17	1.05 (0.43–2.56)	0% (0.59)
Age	3 (230)	82/148	–	− 5.83 (− 9.73, − 1.94)***	–

*Whole brain radiotherapy (resection/biopsy = 19/39); targeted radiotherapy (resection/biopsy = 2/0); no specific data (resection/biopsy = 8/8)

**HD-MTX alone (resection/biopsy = 12/40); HD-MTX-based chemotherapy (resection/biopsy = 169/433); other chemotherapy treatment (resection/biopsy = 2/1); no specific data (resection/biopsy = 23/25)

***Pooled difference of medians (95% CI)

### Overall survival and progression-free survival

**Fig. 2:**
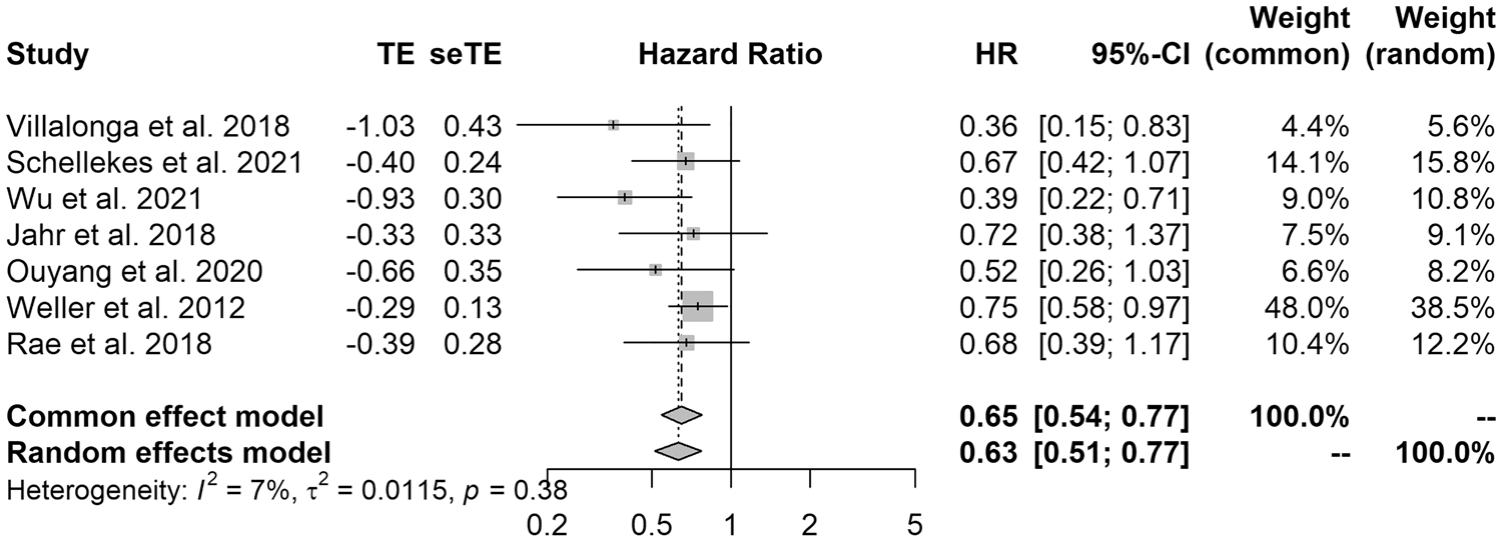
Forest plot showing the overall survival of patients who underwent surgical resection versus biopsy of PCNSL

Seven studies were included in the meta-analysis of survival. The results of the pooled analysis showed that surgical resection was associated with significantly better OS (HR 0.63 [95% CI 0.51–0.77]; I^2^ = 7%) when compared with biopsy (Fig. 2).

**Fig. 3:**
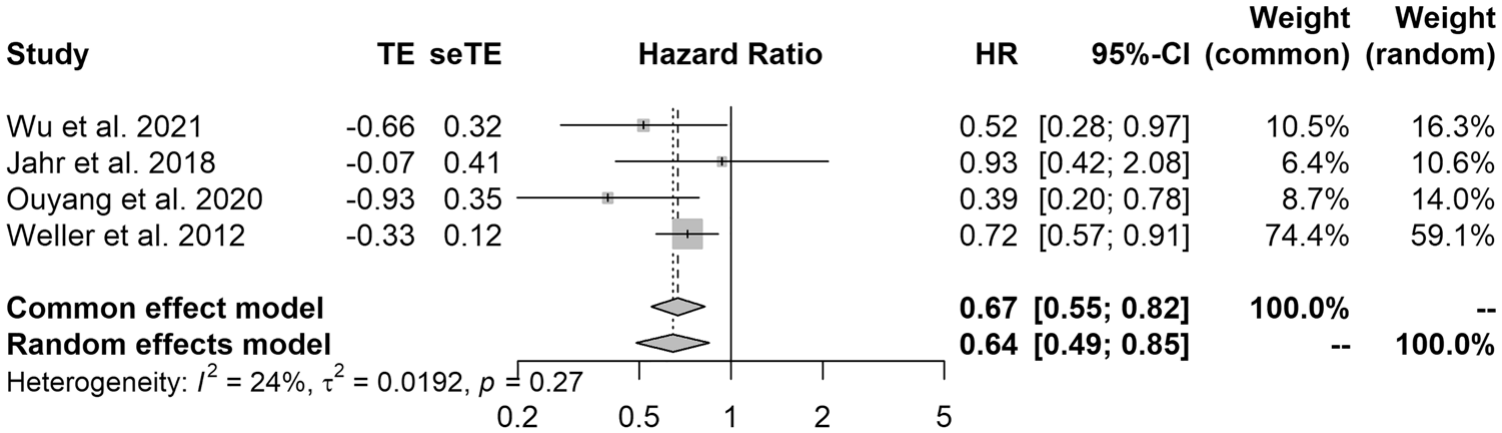
Forest plot showing the progression-free survival of patients who underwent surgical resection versus biopsy of PCNSL

Four studies were included in the meta-analysis of PFS. The results of the pooled analysis showed that resection was associated with significantly better PFS (HR 0.64 [95% CI 0.49–0.85]; I^2^ = 24%) when compared with biopsy (Fig. 3).

### Between-study heterogeneity

Between-study heterogeneity was low or insignificant for most outcomes. The only meta-analysis with substantial heterogeneity (I^2^ = 51%) was the multiple lesion one. In the sensitivity assessment, we found that the study by Wu et al. [25] contributed the most to the heterogeneity; omitting this study reduced heterogeneity to 0%, and the overall effect changed to OR 0.48 (95% CI 0.33–0.71). The main estimates were robust to a leave-one-out analysis, suggesting that they were not driven solely by one study.

## Discussion

This systematic review and meta-analysis included over a thousand patients with PCNSL. We have demonstrated that patients who underwent resection had a significantly lower risk of death and disease progression as compared with patients who underwent biopsy alone. A low or moderate level of heterogeneity in most outcomes strengthened the robustness of the results.

So far, a limited number of studies have examined the impact of surgical resection of PCNSL on overall and progression-free survival. We have found seven studies that addressed this issue and met the inclusion criteria for the meta-analysis. Most of them provided evidence favoring surgical resection over biopsy (at least in specific subgroups of patients with PCNSL) in terms of overall survival and/or progression-free survival. Contrary findings showing no significant difference in OS and PFS between those two treatments have been reported in the study by Jahr et al. [27]. The authors concluded that resection is not recommended as a treatment for PCSNL. However, it is important to note that nearly one-third of their patients were over the age of 70, and more than half of them had multiple lesions and KPS < 70. Moreover, almost 80% of their cohort had deep-seated lesions. The scale designed by Rae et al. [34] (which incorporates age, frailty, superficial vs. deep brain lesion location, and single vs. multiple lesions) indicates that resection does not increase survival for patients in the high surgical risk category. Moreover, in a study of single lesion PCNSL by Schellekes et al. [3] the researchers found that patients below 70 years of age with superficially located lesions significantly benefit from resection (P = 0.007). They also found that survival after resection was improved in a subgroup of patients with a postoperative KPS score of > 70 (P = 0.030).

Involvement of deep brain structures, multiple lesions, low KPS, and advanced age are negative prognostic factors for patients with PCNSL [35–39]. Patients selected for biopsy often have multiple or deep-seated lesions; they are often older and in a worse clinical state than patients selected for resection [3, 24, 25, 40]. We have identified that patients who underwent resection were more likely to have a single, more superficially located lesion than those referred for biopsy. We have also found that those who underwent surgical resection were younger than patients who underwent biopsy alone. This indicates a potential selection bias, which might contribute to the observed differences in the outcomes between the resection group and the biopsy group, confounding the results.

The complication rates for surgical resection and biopsy seem to be comparable [40, 41]. Cloney et al. [40] concluded that surgical resection of PCNSL is safe for selected patients and that the complication rate is comparable to that of other intracranial neoplasms. The studies included in the meta-analysis of complications reported surgical site bleeding, surgical site infection, meningitis, cerebrospinal fluid leak, intracranial bleeding, seizure, focal deficit, brain infarction, and systematic complications [3, 25]. The meta-analysis showed no difference in complications between those who underwent surgical resection and those who underwent biopsy. One caveat to the findings outlined above is the relatively low number of patients analyzed; therefore, future studies investigating the complications associated with surgical resection for PCNSL are warranted.

High-dose methotrexate-based chemotherapy is recommended as an effective first-line treatment for PCNSL [18, 42–44], associated with improved survival [9]. The European Association of Neuro-Oncology (EANO) guidelines for the treatment of PCNSL recommend methotrexate at a high dose (≥ 3 g/m^2^) in rapid, 2–3 h intravenous (iv) infusions for a minimum of 4–6 injections at 2–3 week intervals [18]. The rapid infusion is essential for increasing MTX penetration into the CNS and improving tumor response [45]. Patients in the G-PCNSL-SG-1 study [5] were given six cycles of methotrexate at a dose of 4 g/m^2^ as an iv infusion over four hours, which carried the risk of low MTX concentrations in CSF and decreased tumor response [45], potentially affecting the result of the post hoc analysis by Weller et al. [24] In addition, several recent trials used higher doses of MTX [43, 46, 47]. The results of the study by Li et al. [43] suggest that a dose of 8 g/m^2^ might be beneficial for patients with PCNSL, although the optimal methotrexate dose has yet to be determined [18, 48]. In comparison to HD-MTX alone, combining it with other chemotherapeutic agents might be beneficial [44, 49, 50].

So far, several studies have been conducted to evaluate the efficacy of radiotherapy in patients with PCNSL. However, the results are still unclear [14]. Recent studies have shown that adding adjuvant radiotherapy to surgery improves survival [21]. In the present study, we found no differences between the resection group and the biopsy group in terms of the use of postoperative radiotherapy.

To date, one systematic review evaluating resection versus biopsy in patients with PCNSL has been published. In the study by Labak et al. [51], the authors included 24 articles, of which 15 failed to show benefit from resection. However, most of the studies that failed to show the superiority of surgery over biopsy were published in the previous century. In the present study, only studies published after 2000 were included.

We have identified three national datasets during literature screening evaluating the role of surgical resection of PCNSL. Two of them indicated the superiority of craniotomy over biopsy regarding survival [22, 34]. In the study by Rae et al. [34], the authors found that, independently of subsequent radiotherapy and chemotherapy, craniotomy was associated with increased median survival compared with biopsy; furthermore, gross total resection was associated with better survival than biopsy. The third dataset found no evidence in favor of resection. The authors reported that tumor resection had no effect on prognosis [52].

Nationwide dataset studies, however, have some important limitations that we tried to overcome in this study. First and foremost, there is a possibility of coding mistakes, which means that codes for craniotomies might be used for biopsy as “glorified biopsies”, instead of resections, which might have affected the overall findings [34]. Second, in large datasets, missing data is an inherent limitation. In the present study, most of the included studies were single-center. We believe such studies are less susceptible to these limitations. Furthermore, nationwide datasets such as SEER and NCDB are limited to the United States, limiting the findings’ generalizability [34]. In this study, we included patients from seven countries.

However, our study has some limitations. First, database searching was limited by English-language restrictions. This might result in omitting potentially relevant studies in other languages, introducing language bias. Second, the majority of the included studies were single-center retrospective cohorts, with all their inherent limitations, including selection bias. Most of the included studies lacked data on performance status, postoperative complications, and other clinical and tumor features stratified by surgical approach (resection or biopsy). Similarly, not all studies provided detailed information on chemotherapy and/or radiotherapy. Additionally, we could not control for several potential confounding factors because of the lack of data. Furthermore, in most studies, lnHRs were calculated from available data; although using a validated method, the computations could not perfectly reflect the accuracy of the OS and PFS outcomes. Accordingly, the results of the meta-analysis of OS and PFS should be interpreted with caution. Finally, unpublished studies with negative findings may have led to biased results. Following recommendations by Sterne et al. [53], we did not assess publication bias because it may lead to inappropriate and misleading findings if fewer than 10 studies are included.

## Conclusions

In selected patients, surgical resection of PCNSL is associated with significantly better overall survival and progression-free survival compared with biopsy alone. Potential confounders preclude causal conclusions. Due to the observational design of available studies, clinical trials are required to further evaluate the outcomes in a controlled environment.

## Supplementary Information

Below is the link to the electronic supplementary material.

Supplementary file1 (DOCX 15 kb)

## Data Availability

The datasets generated during and/or analysed during the current study are available from the corresponding author on reasonable request.
