# Supplementary material for: Surgical resection versus biopsy in the treatment of primary central nervous system lymphoma: a systematic review and meta-analysis
Source: J Neurooncol. 2022 Nov 30;160(3):753–61. doi: 10.1007/s11060-022-04200-7 (PMC9758097; doi:10.1007/s11060-022-04200-7)
Supplement: Supplementary file 1 — Supplementary file1 (DOCX 15 kb) [file 11060_2022_4200_MOESM1_ESM.docx]

**Title: Surgical resection versus biopsy in the treatment of primary central nervous system lymphoma: a systematic review and meta-analysis**

Authors: Rafał Chojak ^1,2^, Marta Koźba-Gosztyła ^2^ , Karolina Polańska^1^, Marta Rojek^1^, Aleksandra Chojko^1^, Rafał Bogacz^1^, Natalia Skorupa^1^, Jakub Więcław^1^, Bogdan Czapiga ^2,3^

^1^ Faculty of Medicine, Wroclaw Medical University, Wroclaw, Poland

^2^ Department of Neurosurgery, 4th Military Hospital in Wroclaw, Wroclaw, Poland

^3^ Department of Nervous System Diseases, Faculty of Health Sciences, Wroclaw Medical University, Wroclaw, Poland

Corresponding author:
Rafał Chojak

**ORCID: 0000-0002-7644-3444**

Wrocław Medical University

Ludwika Pasteura 1

50-367 Wrocław

Phone: +48 505 239 425
E-mail: [rafalchojak@gmail.com](mailto:rafalchojak@gmail.com)

**Appendix 1.** Literature Search Strategy.

| Scopus (2001-01-01 - 2022-10-01) |
| --- |
| ( TITLE-ABS-KEY ( "primary central nervous system lymphoma" OR "central nervous system lymphoma" OR "pcnsl" OR "cerebral lymphoma" OR "brain lymphoma" ) OR TITLE-ABS-KEY ( ( "diffuse large b-cell lymphoma" ) AND ( "central nervous system" OR "cerebral" OR "brain" ) ) AND TITLE-ABS-KEY ( "resection" OR "surgery" OR "operation" OR "craniotomy" ) ) |

| Web of Science (2001-01-01 - 2022-10-01) |
| --- |
| ((TS=("primary central nervous system lymphoma" OR "central nervous system lymphoma" OR "pcnsl" OR "cerebral lymphoma" OR "brain lymphoma")) OR TS=(("diffuse large b-cell lymphoma") AND ("central nervous system" OR "cerebral" OR "brain"))) AND TS=("resection" OR "surgery" OR "operation" OR "craniotomy") |

| Embase (2001-01-01 - 2022-10-01) |
| --- |
| ('primary central nervous system lymphoma':ti,ab,kw OR 'central nervous system lymphoma':ti,ab,kw OR 'pcnsl':ti,ab,kw OR 'cerebral lymphoma':ti,ab,kw OR 'brain lymphoma':ti,ab,kw OR ('diffuse large b-cell lymphoma':ti,ab,kw AND ('central nervous system':ti,ab,kw OR 'cerebral':ti,ab,kw OR 'brain':ti,ab,kw))) AND ('resection':ti,ab,kw OR 'surgery':ti,ab,kw OR 'operation':ti,ab,kw OR 'craniotomy':ti,ab,kw) |

| PubMed (2001-01-01 - 2022-10-01) |
| --- |
| ("Primary central nervous system lymphoma"[Text Word] OR "central nervous system lymphoma"[Text Word] OR "PCNSL"[Text Word] OR "cerebral lymphoma"[Text Word] OR "brain lymphoma"[Text Word] OR ("diffuse large B-cell lymphoma"[Text Word] AND ("central nervous system"[Text Word] OR "cerebral"[Text Word] OR "brain"[Text Word]))) AND ("resection"[Text Word] OR "surgery"[Text Word] OR "operation"[Text Word] OR "craniotomy"[Text Word]) |
